# Identification of patients with potential palliative care needs: A systematic review of screening tools in primary care

**DOI:** 10.1177/0269216320929552

**Published:** 2020-06-07

**Authors:** Yousuf ElMokhallalati, Stephen H Bradley, Emma Chapman, Lucy Ziegler, Fliss EM Murtagh, Miriam J Johnson, Michael I Bennett

**Affiliations:** 1 Academic Unit of Palliative Care, Leeds Institute of Health Sciences (LIHS), School of Medicine, University of Leeds, Leeds, UK; 2 Academic Unit of Primary Care, Leeds Institute of Health Sciences (LIHS), School of Medicine, University of Leeds, Leeds, UK; 3 Wolfson Palliative Care Research Centre, Hull York Medical School, University of Hull, Hull, UK

**Keywords:** Palliative care, terminal care, mass screening, primary health care, systematic review, advance care planning, symptom assessment, terminally ill

## Abstract

**Background:**

Despite increasing evidence of the benefits of early access to palliative care, many patients do not receive palliative care in a timely manner. A systematic approach in primary care can facilitate earlier identification of patients with potential palliative care needs and prompt further assessment.

**Aim:**

To identify existing screening tools for identification of patients with advanced progressive diseases who are likely to have palliative care needs in primary healthcare and evaluate their accuracy.

**Design:**

Systematic review (PROSPERO registration number CRD42019111568).

**Data sources:**

Cochrane, MEDLINE, Embase and CINAHL were searched from inception to March 2019

**Results:**

From 4,127 unique articles screened, 25 reported the use or development of 10 screening tools. Most tools use prediction of death and/or deterioration as a proxy for the identification of people with potential palliative care needs. The tools are based on a wide range of general and disease-specific indicators. The accuracy of five tools was assessed in eight studies; these tools differed significantly in their ability to identify patients with potential palliative care needs with sensitivity ranging from 3% to 94% and specificity ranging from 26% to 99%.

**Conclusion:**

The ability of current screening tools to identify patients with advanced progressive diseases who are likely to have palliative care needs in primary care is limited. Further research is needed to identify standardised screening processes that are based not only on predicting mortality and deterioration but also on anticipating the palliative care needs and predicting the rate and course of functional decline. This would prompt a comprehensive assessment to identify and meet their needs on time.

**What is already known about the topic?**

- Earlier initiation of palliative care can improve quality of care for individuals with advanced diseases.
- However, disease trajectories are highly variable, so it is difficult to identify the appropriate time to initiate palliative care.
- A systematic approach may help to identify patients with advanced progressive disease and potential palliative care needs who could benefit from holistic assessment.

**What this paper adds?**

- Most screening tools use prediction of death and/or deterioration as a proxy for the identification of people who are likely to have unmet palliative care needs.
- The performance metrics for these tools were generally poor.

**Implications for practice, theory or policy**

- More research is needed to identify a standardised and robust screening tool to identify patients with advanced progressive diseases and potential palliative care needs in primary care.
- Future studies should validate screening tools against an appropriate reference standard, such as palliative care interview to evaluate their ability to identify patients with potential palliative care needs.
- Identification of patients with advanced progressive diseases and potential palliative care needs process should be supported by a comprehensive and holistic assessment to identify their unmet palliative care needs and determine the appropriate care pathway.

## Background

In Europe, 85% of people now die of chronic diseases such as cancer, heart disease, stroke and dementia.^1^ Chronic diseases are characterised by slow progression, fluctuations in trajectory, long duration and uncertainty in prognoses.^2,3^ During advanced stages of chronic life-limiting illnesses, patients usually suffer high levels of pain and other physical and psychological symptoms.^4,5^ At this stage, patients with any progressive disease could benefit from palliative care.^6^

There is evidence from randomised controlled trials that earlier access to specialist palliative care can promote quality of life, reduce hospital length of stay and hospitalisations and even prolong survival.^7–13^ However, current evidence shows that palliative care is often delivered late in the illness trajectory and access to palliative care is inequitable.^14^ In the United Kingdom, around 90,000 people with advanced progressive conditions who could benefit from palliative care are estimated not to be receiving such care every year.^15^

One of the key barriers to providing palliative care on time is the difficulty in identifying patients who could benefit from it.^16,17^ Once the patient is identified as having potential palliative care needs, their needs can be assessed and addressed in a timely manner. However, not all patients with advanced progressive diseases have unmet palliative care needs. In addition, busy healthcare professionals cannot provide holistic assessment for all of these patients.^18^ It has been suggested that a systematic method could facilitate earlier identification of a subset of patients with advanced progressive diseases who are likely to have unmet palliative care needs and hence benefit from palliative care needs assessment.^16,19^

Since most people with chronic diseases live at home in the last phase of their life, primary care teams are in the best position to identify patients with potential palliative care needs who could benefit from palliative care needs assessment.^20–22^ Two systematic reviews have assessed the screening tools that can be used for the identification of patients who are likely to have unmet palliative care needs. However, neither of them examined the accuracy of the available tools.^16,19^ This systematic review aimed to identify the existing screening tools for identification of patients with advanced progressive diseases who are likely to have unmet palliative care needs in primary care and synthesise the available evidence regarding their accuracy.

## Review questions

- What screening tools have been used and studied to identify patients with advanced progressive diseases and potential palliative care needs in primary care?
- What are the main characteristics and differences between these screening tools?
- What is the accuracy of these screening tools?

## Methods

A positivist approach was used to undertake this systematic review and narrative synthesis of the evidence. This research design was selected because the evidence incorporated a wide range of screening tools and included data from different study designs not suitable for a meta-analysis.^23^ The details of the systematic review protocol are provided in PROSPERO (CRD42019111568). The systematic review was conducted and reported following Preferred Reporting Items for Systematic Reviews and Meta-Analyses protocols (PRISMA-P) guidelines.^24^

## Criteria for considering studies for this review

### Types of studies

We included articles that were published in peer-reviewed journals. Commentaries, abstracts, posters, letters to the editor, case reports, reviews and unpublished studies were excluded.

### Types of participants

This review included studies examining adults (18 years or older). Studies that reported mixed populations of children and adults were included if data for adults were reported separately. Only studies which included primary care patients or assessed patients in primary care settings were included. Studies which were conducted in mixed settings were included as long as they included primary care patients.

### Types of intervention

We included studies that mentioned the use or development of any screening tool to identify patients with advanced progressive diseases who are likely to have unmet palliative care needs in primary healthcare. Any type of screening tool (electronic or manual) was considered as long as it has been used to identify primary care patients with potential palliative care needs. We also included studies evaluating the ability of the current screening tools to identify patients who could have unmet palliative care needs.

### Language

The search was restricted to articles reported in the English language.

### Search strategy and study selection

We searched Cochrane Library, MEDLINE, Embase and CINAHL. A search strategy for MEDLINE is presented in Supplementary File 1. Databases were searched from inception to the end of September 2018. The search was updated in March 2019 to include articles published after September 2018. We searched the reference lists of the included studies and the relevant review articles to make sure that all relevant articles were captured. The search strategies were created by one reviewer (YE) and peer reviewed by a librarian and an information specialist, not otherwise associated with the project. The search results were imported into a reference management software package (EndNote X7) to remove duplicated references.

Abstracts of all identified studies were independently screened for inclusion by two reviewers. We obtained the full texts of all abstracts that met the inclusion criteria or where there was insufficient information in the abstract alone to determine eligibility. Final article selection was carried out after reading full papers by two reviewers. Disagreements related to screening were resolved through discussion and where necessary a third researcher was consulted.

### Data extraction

The characteristics of the included studies and screening tools were extracted prior to synthesis. For studies assessing the accuracy of the screening tools, specificity, sensitivity, positive predictive value (PPV) and negative predictive value (NPV) were either extracted from the text or calculated from the reported data. Study authors were contacted to resolve any uncertainties, whenever possible. Data were extracted by one reviewer and double checked for accuracy by a second reviewer.

### Assessing the risk of bias

Two independent reviewers assessed the methodological quality and risk of bias in the studies that examined the accuracy of the screening tools. Disagreements were resolved first through discussion and then by involving a third reviewer for arbitration. For observational studies, we used the Newcastle–Ottawa Scale to assess the risk of bias.^25^ The methodological quality of these studies was rated on a scale from 0 stars to 9 stars. Studies were classified into groups of low (less than 6 stars)-, moderate (7–8 stars)- and high (9 stars)-quality studies. The quality of randomised clinical trials (RCTs) was assessed using the Cochrane Collaboration’s tool for assessing the risk of bias.^26^ This tool evaluates seven possible sources of bias. For each individual domain, studies were classified into low, unclear and high risk of bias.

### Strategy for data synthesis

A narrative synthesis was used with information provided in the tables and text to describe and summarise the main findings and features of the included studies and the identified screening tools.

## Results

### Selection of studies

**Figure 1.**
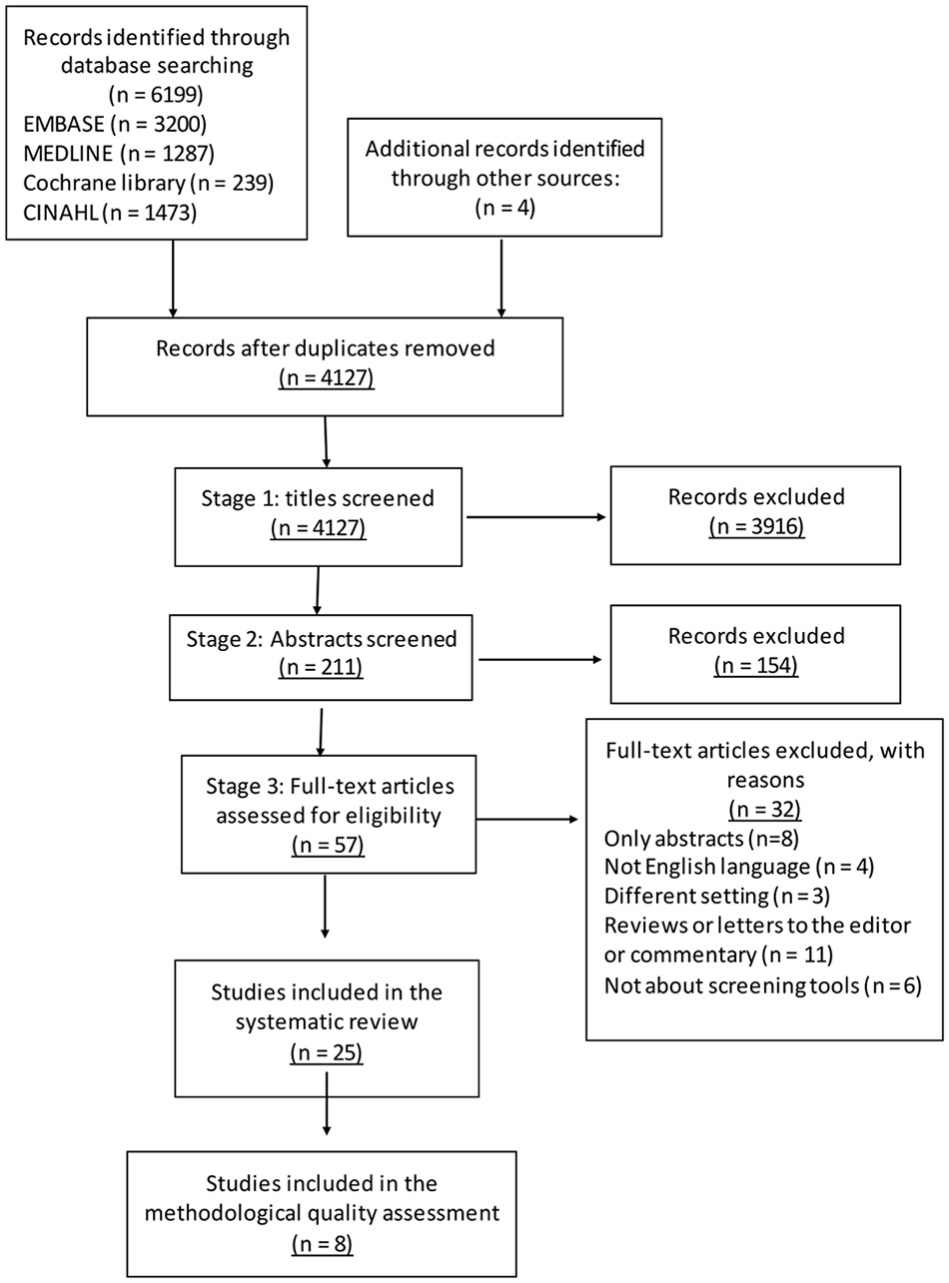
PRISMA flowchart of the study selection.

We identified 6,203 records through the database search and other sources (Figure 1). Of these, 2,076 duplicates were removed, leaving 4,127 publications for title and abstract screening. Fifty-seven articles remained following the review of title and abstract. An additional 32 articles were excluded following full-text review, resulting in a total of 25 articles. Of these, only eight evaluated the accuracy of screening tools. No studies were excluded based on their quality assessment.

### Characteristics of the included studies

The main characteristics of the 25 articles included in the review are outlined in Table 1.^20,21,27–49^ Most studies were published within the last 5 years (2015–2019). Of those, 17 studies were carried out in the United Kingdom (7), the Netherlands (6) and Spain (4). Twelve studies were observational (prospective observational and cross-sectional), nine studies incorporated mixed methods, three studies were RCTs and one was a service evaluation study. The majority of the studies included patients with a variety of both cancer and non-cancer conditions. A total of 17 studies were conducted exclusively in primary care settings and the remaining studies in mixed settings, including primary care.

**Table 1.** Characteristics of the included articles.

Tool	Reference	Country	Setting	Study design	Study objectives	Population(s) tested in (final sample size)	Rating by	Percent of patients identified by ST
SQ^a^	Barnes et al.^45^	UK	Primary care	Prospective observational study	To identify predictive factors of mortality for heart failure patients in primary care, and to report the sensitivity and specificity of prognostic information from GPs.	Patients with heart failure > 60 y (231)	GPs	41.1%
SQ^a^	Moroni et al.^44^	Italy	Primary care	Prospective cohort study	To determine the prognostic accuracy of GPs asking the SQ about their patients with advanced cancer.	Advance cancer patients (231)	GPs	54.6%
SQ^a^	Lakin et al.^46^	USA	Primary care	Retrospective observational study	To assess the SQ performance in primary care setting.	Patients screened for a high-risk care management programme in primary care (1,737)	GPs	6.6%
GSF PIG^a^	Clifford et al.^28^	UK	Primary care	Service evaluation	To describe the most recent developments and outline the potential of the updated version of GSF Gold Programme.	Primary care patients	–	–
GSF PIG (Italian version)^a^	Scaccabarozzi et al.^27^	Italy	Primary care and home palliative care units	Prospective observational study	To demonstrate the characteristics of patients with palliative care needs, who early identified by GPs and to explore their care process in home palliative care services.	Primary care patients (139,071)	GPs	0.67%
SPICT (German version)^a^	Afshar et al.^29^	Germany	Primary care	Mixed methods	To develop, refine and evaluate SPICT (German version) for its application in primary care.	Primary care patients (case vignettes)	GPs	–
SPICT (Japanese version)^a^	Hamano et al.^30^	Japan	Primary care	Cross-sectional study	To identify the prevalence and characteristics of primary care patients being at risk of deteriorating and dying, as determined by SPICT.	Adults > 65 y (382)	GPs	17.3%
SPICT (Japanese version)^a^	Hamano et al.^20^	Japan	Primary care	Cross-sectional study	To explore the prevalence and characteristics of family practice patients who need palliative care approach as determined using supportive and palliative care indicators tool.	Adults > 65 y (87)	GPs	9.2%
SPICT^a^	Highet et al.^32^	UK	Primary care and hospitals	Mixed methods	To refine and test SPICT tool to help multidisciplinary teams, to identify patients at risk of deteriorating and dying in all care settings.	Patients with advanced organ failure	Physician and nurse	–
SPICT (Spanish version)^a^	Fachado et al.^31^	Spain	Primary care and socio-sanitary services	Mixed methods	To translate, cross-culturally adapt to Spanish and evaluate the Spanish version of the SPICT.	Patients with advanced progressive diseases (188)	Physician and nurse	–
SPICT (2012 version) and SQ^a^	Mitchell et al.^36^	Australia	Primary care	RCT	To test whether screening for likely death within 12 months using SPICT and SQ Is more effective than an intuition approach.	Adults > 70 y (4,365)	GPs	11.7% (SQ) 5.1% (SPICT)
NECPAL & SQ^a^	Gómez-Batiste et al.^34^	Spain	Primary care, hospitals, social health centres and nursing homes	Prospective cohort study	To investigate the predictive validity of the NECPAL and SQ to determine 12- and 24-month mortality.	Patients with advanced chronic conditions and limited life prognosis (1,059)	GPs and nurse	79% (SQ) 73.7% (NECPAL)
NECPAL^a^	Gómez-Batiste et al.^33^	Spain	Primary care, hospitals, social health centres and nursing homes	Cross-sectional study	To determine the prevalence of advanced chronically ill patients limited life prognosis in need of palliative care using NECPAL tool.	Primary care patients (51,595)	GPs and nurse	1.6% (SQ) 1.5% (NECPAL)
NECPAL^a^	Gómez-Batiste et al.^35^	Spain	Primary care, hospitals, social health centres and nursing homes	Mixed methods	To develop the NECPAL tool to identify patients in need of palliative care.	Patients with advanced chronic diseases (1,059)	GPs and nurse	–
RADPAC^a^	Thoonsen et al.^38^	The Netherlands	Primary care	Cross-sectional study after RCT	To examine whether trained GPs identified more patients in need of palliative care using RADPAC tool and provided multidisciplinary care more than untrained GPs.	Primary care patients (6,278)	GPs	–
RADPAC^a^	Thoonsen et al.^39^	The Netherlands	Primary care	RCT	To train GPs in identifying patients in need of palliative care and in structuring anticipatory palliative care planning and studied its effect on the quality of life.	Primary care patients	GPs	–
RADPAC^a^	Thoonsen et al.^37^	The Netherlands	Primary care	Mixed methods	To develop a tool for identification of patients with congestive heart failure, COPD and cancer who could benefit from proactive palliative care in primary care.	Primary care patients	GPs	–
PALLI^a^	Vrijmoeth et al.^41^	The Netherlands	Primary care, central residential settings and intellectual disability physician clinics	Mixed methods	To evaluate feasibility, construct validity and predictive validity of PALLI.	Patients with intellectual disability who were more likely to be in need of palliative care (190)	GPs, intellectual disability physician and daily care professionals	–
PALLI^a^	Vrijmoeth et al.^40^	The Netherlands	Primary care, central residential settings and intellectual disability physician clinics	Mixed methods	To describe development of PALLI and to explore its applicability.	Patients with intellectual disability who were more likely to be in need of palliative care (190)	GPs, intellectual disability physician and daily care professionals	–
The double SQ^a^	Weijers et al.^49^	The Netherlands	Primary care	Pilot RCT with caged vignettes	To pilot test whether adding SQ2 to SQ1 prompts GPs to plan for anticipatory palliative care.	Case vignettes (primary care patients)	GPs	–
Raincine tool^b^	Rainone et al.^21^	USA	Primary care	Prospective observational study	To develop a methodology to identify patients who may benefit from palliative care and provide estimates of their prevalence in primary care.	Primary care patients (18,308)	Electronic tool	4.6%
AnticiPal (updated version)^b^	Mason et al.^43^	UK	Primary care	Mixed methods	To refine and evaluate the utility of an electronic ST to help primary care teams screen their patients for people who could benefit from palliative care.	Primary care patients (62,708)	Electronic tool	0.61%–1.23% (0.8% for all practices)
AnticiPal^b^	Mason et al.^42^	UK	Primary care	Mixed methods	To develop and test an electronic ST in primary care as a tool to improve patient identification for a palliative care approach.	Primary care patients (83,229)	Electronic tool	0.6–1.7%
eFI^b^	Stow et al.^48^	UK	Primary care	Longitudinal population-based study (case control study)	To identify frailty trajectories that could indicate increased risk of dying and the need to consider palliative care.	Adults > 75 y (26,298)	Electronic tool	1.1%
eFI^b^	Stow et al.^47^	UK	Primary care	Prospective case control study	To examine if changes in eFI could indicate whether individuals are at increased risk of mortality and may require palliative care.	Adults > 75 y (13,149)	Electronic tool	0.49%

COPD: chronic obstructive pulmonary disease; GSF PIG: gold standard framework proactive identification guidance; SPICT: the supportive and palliative care indicators tool; NECPAL: Necesidades Paliativas [Palliative Needs]; SQ: surprise question; eFI: Electronic Frailty Index; GPs: general practitioner; PALLI: PALliative care: learning to identify in people with intellectual disabilities; palliative care: palliative care; ST: screening tool; RCT: randomised control trial.

a Paper-based screening tools.

b Electronic tools.

### Characteristics of the screening tools

Ten screening tools, used to identify patients with advanced progressive diseases who are likely to have unmet palliative care needs, were identified in this systematic review. Of these, nine were originally designed to identify patients with potential palliative care needs and one was originally developed to identify patients with frailty (Table 2). Four tools were originally developed in the United Kingdom (Gold Standard Framework–Proactive Identification Guidance: GSF PIG, Supportive, and Palliative Care Indicators Tool: SPICT, AnticiPal electronic tool, and Electronic Frailty Index: eFI), three in the Netherlands (RADboud indicators for PAlliative Care Needs: RADPAC, PALliative care: Learning to Identify in people with intellectual disabilities: PALLI, and the double Surprise Question(SQ)), two in the United States (SQ and early identification tool for palliative care patients ‘Rainoe tool’), and one tool in Spain (Necesidades Paliativas [Palliative Needs]: NECPAL tool). Seven of the identified tools were paper-based screening tools and three of them were electronic case finding tools. The screening object for most of the identified tools was to identify patients who are at a high risk of deteriorating and dying and might benefit from palliative care. The time frame within which symptoms and clinical indicators are assessed varies across the screening tools. The PALLI tool assesses the health status over the last 3–6 months, but the time period for assessment is unspecified for the majority of the symptoms and clinical indicators in all other screening tools. Reviewing care, assessment of needs and initiating discussions about end-of-life needs are some examples of the recommended actions following the screening (Table 3).

**Table 2.** Summary of the main features of tools which were designed to identify patients with potential palliative care needs.

Tool^a^	Screening objectives	Languages	Target population	Setting (Primary care/GP, Hospital)	Type: paper-based/electronic tool	Completion time	Time frame of assessment	Cutoff value	Actions taken following screening
SPICT	To identify people who are at risk of deteriorating and dying and might benefit from palliative care.	English. Japanese, German, Spanish	All	Primary care/GP, hospital	Paper-based	SPICT: few minutes. SPICT-DE: an average of 7.5 min. SPICT-ES: an average of 4 min and 45 s.	Unspecified for most variables	SPICT 2019 version), SPICT-DE and SPICT-ES: no cutoff value. SPICT-J: SPICT + (⩾2 general indicators or ⩾1 clinical indicator). SPICT-ES: SPICT + (⩾2 general indicators and ⩾1 clinical indicator).	Review current care and care planning (e.g. review current treatment and consider referral for specialist assessment if symptoms are complex).
NECPAL	To Identify people who are at high risk of dying (who likely in need of palliative care).	Spanish	All	Primary care/GP, hospital	Paper-based	NM (one page)	Unspecified for most variables	NECPAL + (SQ+, and ‘⩾1 general indicator or ⩾1 specific indicator’).	Consider actions such as a holistic assessment, review of treatment and advance care planning.
RADPAC	To identify people who could benefit from palliative care based on their clinical indicators.	Dutch	COPD, congestive heart failure and cancer patients	Primary care/GP	Paper-based	NM (one page)	Unspecified for most variables	No cutoff point	Discuss with patient and their family to explore their problems needs ‘proactive palliative care planning’.
GSF PIG	To identify people who may be in their final stage of life who could benefit from an early palliative approach.	English, Italian	All	Primary care/GP, hospital	Paper-based	NM (one page)	Unspecified for most variables	GSF PIG + (SQ+, ⩾1 general indicator or ⩾1 specific indicator).	Assess needs through advance care planning, discussions and plan care tailored to patient choices.
PALLI	To identify patients with intellectual disability who may benefit from palliative care via screening deteriorating health, indicative of a limited life expectancy.	Dutch	Patients with intellectual disabilities	Primary care/GP	Paper-based	Mean time of 10.5 min (physicians) and 10.1 min (daily care professionals)	Previous 3–6 months for all domains except fragility	No cutoff point	Discuss with patients their health status and their need for palliative care in a multidisciplinary setting.
SQ	To identify patients with poor prognosis who might benefit from palliative care.	English, Italian	All	Primary care/GP, hospital	Paper-based	NM (one question)	NA	SQ+ (answer no to the ‘surprise’ question).	Initiate discussions about end-of-life needs and preferences.
The double SQ	To identify patients with poor prognosis who might benefit from palliative care.	Dutch, Slovak	All	Primary care/GP, hospital	Paper-based	NM ( two questions)	NA	The double SQ+ (a combination of SQ1: ‘no’ and SQ2: ‘yes’).	Prompt GPs to plan for anticipatory palliative care.
AnticiPal	To identify patients who potentially have deteriorating health due to one or more advanced illnesses and a likelihood of unmet supportive and palliative care needs.	English	All	Primary care/GP	Electronic	NA	Unspecified for most variables. Previous 18 months for Codes that indicate malignancy	AnticiPal+ (if one or more inclusion criteria are met, none of the exclusion criteria is met). The inclusion criteria: Type 1: Malignancy codes, e.g. pancreatic cancer. Type 2: Other single Read Codes at any time, e.g. Frailty. Type 3: Combinations of Read Codes, e.g. difficulty swallowing and dementia.	Create a list of patients for review and care planning.
Racine tool	To identify people who are at high risk of death (who may benefit from palliative care).	English	All	Primary care/GP	Electronic	NA	NA	Patient is included if their electronic records contained at least one of the marker for high risk of death within the next year, e.g. age > 75 or a diagnosis of congestive heart failure.	Create a preliminary screen to assist clinicians in early identification of patients in needs of palliative care.

COPD: chronic obstructive pulmonary disease; GSF PIG: gold standard framework proactive identification guidance; SPICT: the supportive and palliative care indicators tool; NECPAL: Necesidades Paliativas [Palliative Needs]; SQ: surprise question; GPs: general practitioner; PALLI: palliative care: learning to identify in people with intellectual disabilities; NM: not mentioned; NA: not applicable; +: positive.

a The most recent version of the tool.

**Table 3.** Summary of the general and specific indicators of deteriorating health and increasing needs in the tools that were designed to identify patients with potential palliative care needs.

	GSF PIG	SPICT	NECPAL	RADPAC	AnticiPal	PALLI
SQ	Yes	No (SQ was part of some previous versions of SPICT but was removed from the recent versions of SPICT in different languages)	Yes	No	NA	Yes
Nutritional decline	Progressive weight loss (>10%) in the past 6 months. Serum albumen < 25 g/l	Progressive weight loss or remains underweight. Low muscle mass.	Weight loss > 10%	NM	NM	Weight loss
Functional decline	In bed or chair 50% of the day. General physical and performance status decline (Barthel score) and decreasing activities. Increasing dependence and need for support.	In bed or chair > 50% of the day. Poor or deteriorating performance status. Dependent and increasing need for support.	– Karnofsky or Barthel score > 30% loss of two or more activities of the daily living. Severe dependence (Karnofsky < 20).	NM	Codes that indicate housebound. Codes that indicate very poor mobility	Spending more time in bed. Less able to perform activities in the daily living (ADL). General physical decline.
Cognitive decline	–	NM	Minimental/Pfeiffer Decline	NM	NM	Cognitive deterioration (e.g. remembers less, less oriented)
Symptom burden	Unstable, deteriorating, complex symptom burden	Persistent symptoms despite optimal treatment	Persistent symptoms (e.g. pain, weakness, anorexia, dyspnoea, digestive)	NM	NM	Having more severe symptoms (progressive)
Psychosocial decline	NM	NM	Present of emotional stress (Detection of Emotional Distress Scale (DME) > 9). Severe Social Vulnerability (social and family assessment).	NM	NM	Restless behaviour, depression, stress
Multi-morbidity	Significant multi-morbidities	NM	>2 chronic diseases	NM	Codes that indicate multiple organ failure and multimorbidity	Other serious chronic conditions (in addition to intellectual disability)
Urgent/unplanned admissions	Repeated unplanned hospital admissions	Unplanned hospital admission(s)	>2 urgent or not planned admittances in last 6 months	NM	NM	NM
Presence of an adverse event	Sentinel event, e.g. serious fall, bereavement, transfer to nursing home	NM	Geriatric syndromes (at least two): Falls, pressure ulcers, dysphagia, delirium, recurrent infections	NM	NM	Recurrent infections
Others	Considered eligible for DS 1500 payment^a^					Geriatric home admission exam
Choice of no further active treatment/ no curative treatment available	Choice for no further active treatment	Chooses to reduce, stop or not have treatment (patient or family)	Limitations of therapeutic effort were mentioned by patient, family or the team	NM	NM	Any serious chronic conditions that cannot be treated or which continued treatment is not indicated.
Choosing or requiring palliative care	Asks for palliative care by patient	Asks for palliative care by patient or family	Asks for palliative care by patient, family or the team	NM	NM	NM
Additional specific clinical indicators for	Cancer, heart disease, COPD, kidney disease, liver disease, general neurological diseases, Parkinson’s disease, motor neurone disease, multiple sclerosis, frailty, dementia, stroke	Cancer, heart/ vascular disease, kidney disease, liver disease, neurological disease, respiratory disease, dementia/ frailty	Cancer, COPD, chronic heart disease, chronic neurological disease (CVA, ALS, motor neurone disease, multiple sclerosis), dementia	COPD, congestive heart failure and cancer	Cancer, heart/vascular disease, kidney disease, liver disease, dementia, frailty, stroke	Intellectual disability and frailty

COPD: chronic obstructive pulmonary disease; GSF PIG: gold standard framework proactive identification guidance; SPICT: the supportive and palliative care indicators tool; NECPAL: Necesidades Paliativas [Palliative Needs]; SQ: surprise question; PALLI: PALliative care: Learning to Identify in people with intellectual disabilities; NM: not mentioned; NA: not applicable.

a DS 1500 is a Form for patients who are terminally ill who are not expected to live for more than 6 months to rapidly access benefits in the United Kingdom.

Table 3 summarises the general and specific indicators of the screening tools for identification of people with potential palliative care needs in primary care. The SQ is part of all of the paper-based tools (except the RADPAC and the current versions of SPICT). Five tools (GSF PIG, SPICT, NECPAL, PALLI and AnticiPal) contain general indicators for decline and increasing needs such as repeated unplanned hospital admissions, progressive weight loss and functional decline. Only NECPAL and PALLI contain indicators for psychological and cognitive decline. Six tools (GSF PIG, SPICT, NECPAL, RADPAC, PALLI and AnticiPal) contain additional disease-specific clinical indicators of decline for a number of medical conditions. In the paper-based tools, the number of items or questions varied significantly and ranged from 1 to 42. The remainder of this section describes the included tools which used to identify patients who may benefit from palliative care in primary care.

1. The SQ, which was originally developed by Lynn, is the first tool that has been used for this purpose.^36,50^ It is utilised as a part of some screening tools or used in isolation. The SQ asks whether the respondent would be surprised if the patient died within a specified time period (usually the next year). The SQ has been widely validated in different settings.^34,36,44^ The proportion of patients identified by SQ as having potential palliative care needs across studies ranged from 1.6% to 79%. In those studies applied to patients with advanced progressive diseases, the percentage of patients identified by SQ as having potential palliative care needs ranged from 41% to 79%, whereas that applied SQ to more general populations reported percentages between 1.6% and 11.7%.^34,36,44^
2. The double SQ was developed by adding an additional question (SQ2) that asks whether the respondent would be surprised if the patient is still alive after 12 months when SQ1 is answered in the negative.^49^ The purpose of adding the second SQ was to increase the predictive value of SQ1. The validity of this tool has not been explored yet, although a pilot study concluded that the majority of GPs considered it a useful addition to SQ1.^49^
3. The GSF PIG was developed in the United Kingdom.^27,28^ The tool, which is applicable across care settings, uses the SQ, along with general and disease-specific indicators of decline and increasing need. To the authors’ knowledge, there is no underlying research about the development of GSF PIG, and no validation studies have been performed in primary care settings in the United Kingdom. The GSF PIG has been translated and adapted for the Italian context.^27^ An Italian study which utilised the GSF PIG among primary care patients found that 0.67% of the patients identified as having a low life expectancy, and palliative care needs.^27^
4. The SPICT was developed in the United Kingdom using a process of literature review, peer review and a prospective case-finding study.^32^ It is a one-page tool which consists of a combination of general indicators of deteriorating health and disease-specific indicators. The SPICT had been translated and adapted to Japanese, German and Spanish settings.^20,29,31^ These translated versions (in addition to the original English version) have been validated in a wide range of inpatient and outpatient clinical settings.^20,29,31,32,36^ Various cutoff scores were used in different versions of SPICT (Table 3). Studies in Australia and Japan that used SPICT among old patients in primary care showed that between 5.1% and 17.3% of these patients could benefit from palliative care.^20,30,36^
5. Mason et al.^42^ developed an electronic tool called AnticiPal based on the SPICT criteria. This electronic tool was developed initially through an iterative process of designing, implementation and testing. In a recent study to evaluate the utility of AnticiPal in Scotland, around 0.8% of 62,708 registered patients at eight GP practices were identified as having potential palliative care needs.^43^
6. The NECPAL tool was developed in Catalonia, Spain based on SPICT and the GSF PIG tools.^35^ This instrument, the NECPAL, is a checklist which combines the SQ with general clinical indicators of severity and progression (e.g. co-morbidity and resource use); and specific indicators for some medical conditions. NECPAL has been validated in a wide variety of care settings.^15,33–35^ Recent Spanish observational studies which conducted in multiple setting including primary care settings found that 1.5% of primary care patients and 73.7% of patients with advanced progressive diseases met the NECPAL criteria and could benefit from palliative care.^33,34^
7. The RADPAC tool was developed in the Netherlands through a three-step process comprising a literature search, focus group interviews and a Delphi study with GPs.^37^ The RADPAC tool contains specific indicators for congestive heart failure, chronic obstructive pulmonary disease (COPD) and cancer, although it does not include the SQ or general clinical indicators that can be applied to all patients. A Dutch RCT on the effects of training GPs in early identification of patients who could benefit from palliative care using the RADPAC tool did not find any differences between the intervention and control groups in out-of-hours contacts, contacts with their GP, hospitalisations and place of death.^39^ The study also revealed that only one in four patients who died had been identified as in need of palliative care.
8. The PALLI tool was designed to be used to identify people with intellectual disabilities who may benefit from palliative care.^40^ The tool was developed in the Netherlands using five-stage mixed methods design including retrospective survey, interviews, draft version, focus groups and finalisation for testing in practice. This tool, which consists of 39 questions, composed of eight main themes such as physical decline, changes in characteristic behaviour, and increases in symptom burden. The PALLI tool has been validated for use among patients with intellectual disabilities in different settings, including primary care.^49^ PALLI tool shows promising construct validity and feasibility. There is, however, less and mixed evidence for the predictive validity of this tool.^49^
9. Rainoe et al.^21^ used computerised electronic records to identify the most common factors associated with death within the next year among hospitalised patients. A list of the identified factors (including age 75 and over and having diseases, such as heart failure and COPD) was used to identify people who may benefit from palliative care. The electronic tool has been validated against clinical assessment in an observational study in the United States, which found that 5.6% of primary care patients could benefit from palliative care.^21^
10. Electronic Frailty Index (eFI) was developed in the United Kingdom to identify elderly patients in primary care who may be living with frailty.^47^ The eFI uses a ‘cumulative deficit’ model to calculate a frailty score based on a range of deficits, which can be symptoms, signs, diseases and abnormal laboratory test values. The eFI has been used in two recent studies to identify people who are at an increased risk of mortality and may need palliative care.^47,48^ Initially, Stow et al.^47^ examined the ability of eFI to predict mortality by measuring it at a single time point, which found that 1.1% of individuals age 75 and over could benefit from palliative care. Stow et al.^48^ conducted another study using eFI to examine if changes in frailty index can be used to predict mortality and the need to palliative care. The study identified a distinct frailty trajectory which can be used to identify people who are at a higher risk of dying within 12 months. This study found that 0.49% of people age 75 and over were identified as potential candidates for palliative care. The predictive validity of eFI to identify patients with potential palliative care needs has been evaluated in both studies.^47,48^

### Accuracy of screening tools

Eight studies reported accuracy data for five screening instruments (SPICT, SQ, NECPAL, eFI and early identification tool for palliative care patients ‘Rainoe tool’).^21,34,36,44–48^ Reference standards (i.e. the comparator against which the tool was compared) varied across these studies, including 3-month mortality, 12-month mortality, 24-month mortality and clinical assessment. One study was excluded as data were available only on positively screened patients.^27^ Table 4 shows a summary of the sensitivity, specificity, PPV and NPV for the screening tools.

**Table 4.** Summary of the sensitivity, specificity, PPV, NPV value for the screening tools.

Reference	Length of Follow-up	Comparison	Tool	Cutoff value	Reference standard	Final sample (n)	Age, mean or median (SE, SD, range)	Sensitivity (%)	Specificity (%)	PPV (%)	NPV (%)
Mitchell et al.^36^	12 months	Intuition	SPICT (2012 version)	SPICT+ (SQ+ with ⩾2 general indicators or ⩾1 clinical indicator)	12-month mortality	1,525	79.1, mean (SD 6.9)	34.0	95.8	20.5	97.9
			SQ	SQ+ (answer no to the ‘surprise’ question)	12-month mortality			33.7	95.6	14.0	98.4
Gómez-Batiste et al.^34^	24 months	No	NECPAL	NECPAL+ (SQ+, and ‘⩾1 general indicators or ⩾1 specific indicators’)	12-month mortality	1,059	81.3, mean (SD 11.8)	91.3	32.9	33.5	91.0
					24-month mortality			87.5	35.0	45.8	81.7
			SQ	SQ+ (answer no to the ‘surprise’ question)	12-month mortality			93.7	26.4	32.0	91.9
					24-month mortality			91.4	28.7	44.6	84.2
Rainone et al.^21^	6 months (the length of the study)	No	Raincine tool	Patient is included if their electronic records contained at least one of the marker for high risk of death within the next year, e.g. age > 75 or a diagnosis of congestive heart failure	Clinical assessment	18,308	–	94.0	97.0	36.0	99.0
Barnes et al.^45^	12 months	No	SQ	SQ+ (answer no to the ‘surprise’ question)	12-month mortality	231	77, median (range 71–82)	79.0	61.0	11.6	97.8
Moroni et al.^44^	12 months	No	SQ	SQ+ (answer no to the ‘surprise’ question)	12-month mortality	231	70.2 mean (SE 0.9)	69.3	83.6	83.8	69.0
Lakin et al.^46^	12 months	No	SQ	SQ+ (answer no to the ‘surprise’ question)	12-month mortality	1,737	65, mean	20.5	94.4	20.2	94.5
Stow et al.^48^	12 months	No	eFI	People with rapidly rising frailty (initial increase of 0.022 eFI per month before slowing from a baseline eFI of 0.21)	12-month mortality	26,298	For cases: 85.14, mean(SD 5.98) For control: 85.65, mean(SD 5.98)	3.2	99.1	19.8	93.3
Stow et al.^47^	3 months	No	eFI	eFI cut value > 0.19	3-month mortality	7,890	For cases: 85.1, mean(SD 6.0) For control: 85.6, mean(SD 6.0)	76.0	53.0	11.0	97.0

SPICT: the supportive and palliative care indicators tool; NECPAL: Necesidades Paliativas [Palliative Needs]; SQ: surprise question; eFI: electronic frailty index; SE: standard error; SD: standard deviation; PPV: positive predictive value; NPV: negative predictive value; +: positive.

Across all screening tools and studies, only one study had a PPV over 50% (83.8%). The NPV was high for most tools and varied from 99% to 69%. The sensitivity and specificity values varied considerably and ranged from 3.2% to 94% and 26.4% to 99%, respectively. Studies enrolling participants with advanced progressive diseases reported high sensitivity values; however, studies that targeted a general population of primary care (e.g. adults aged 70 and over) reported lower sensitivity values.

### Methodological quality of studies that reported accuracy data for screening tools

The assessment of the risk of bias is summarised in Supplementary File S2 (RCTs), Supplementary File S3(a) (cohort studies) and Supplementary File S3(b) (case control studies). On the basis of the Newcastle–Ottawa scale, three of the five cohort studies were judged to bear a moderate risk of bias (fair quality)^34,45,46^ and one cohort was judged to have a high risk of bias (low quality) due to the lack of description of the follow-up and no adjustment for confounders.^21^ Only one cohort study fulfilled most of the Newcastle–Ottawa scale criteria and had a low risk of bias (high quality).^44^ The Newcastle–Ottawa scale assessment revealed that the two case control studies were all of a fair quality.^47,48^ Based on the Cochrane risk of bias tool, the overall risk of bias for the included RCT was high because of unclear allocation concealment and differential drop-out rates between the two groups.^36^

## Discussion

### Main findings

We identified 10 screening tools for identification of patients with advanced progressive diseases who are likely to have unmet palliative care needs in primary care which varied in content and accuracy, and in general, the validation studies were of low quality and with high risk of bias.

Most of the identified tools use either prediction of death or deterioration or both as proxies for the identification of people who are likely to have unmet palliative care needs. Patients with advanced progressive diseases experience different trajectories of decline and usually have varying needs at different phases in the illness trajectory.^51,52^ Therefore, the identification process should not be based solely on predicting mortality or survival, but it should also focus on anticipating their needs whenever they occur, and predicting the rate and course of functional decline in order to trigger holistic assessment and make a proactive palliative care plan.

The proportion of patients identified with potential palliative care needs across studies ranged from 0.49% to 79%. The accuracy of five tools (of which data were available in eight studies) showed sensitivity ranging from 3.2% to 94%, and specificity ranging from 26.4% to 99%. The wide variation in the accuracy of the screening tools may be caused by both variations in diagnostic groups and disease trajectory during the last year of life.

### Strengths and weaknesses/limitations of the study

This is the first systematic review to assess the evidence on accuracy of screening tools for identification of patients with advanced progressive diseases who are likely to have unmet palliative care needs in primary care. We used a broad search strategy to identify all potentially relevant studies by searching Cochrane Library, MEDLINE, Embase and CINAHL, and the quality of the validation studies was assessed by two reviewers independently with disagreements resolved by a third reviewer.

Our findings are limited by several issues. First, our search strategy was designed to capture all of the relevant papers but given the nature of this topic, it is possible that some papers may have been missed. Although we conducted a comprehensive and broad search of the literature, we only included English language studies. We did not also include unpublished results or studies from the grey literature, which may have introduced publication bias. However, the methodological quality of grey literature is usually lower than the quality of published studies literature.^53,54^ Second, there is no current consensus about a reference standard against which the accuracy of a screening tool could be assessed. All studies used mortality as a reference standard, with the exception of one study that used clinical judgement to determine whether the identified patient could benefit from palliative care.^21^ This is a major flaw in the evidence, in that we know palliative care needs do not relate particularly closely to time to death, especially for some illnesses such as organ failures. Data were universally missing on how many patients identified (or missed) by the screening tools actually had palliative care needs and so we cannot be certain of the true clinical value of these tools.

### What this study adds

Improving identification of patients who are likely to have unmet palliative care needs is a crucial step to overcome inequity in access to palliative care and to ensure that patients receive the right care at the right time to meet their needs and preferences.^55,16^ Identification does not mean referral to specialist palliative care services is necessarily needed, but rather, it should trigger a comprehensive and holistic assessment of palliative care needs of the identified patients and their families.^17,50^

**Figure 2.**
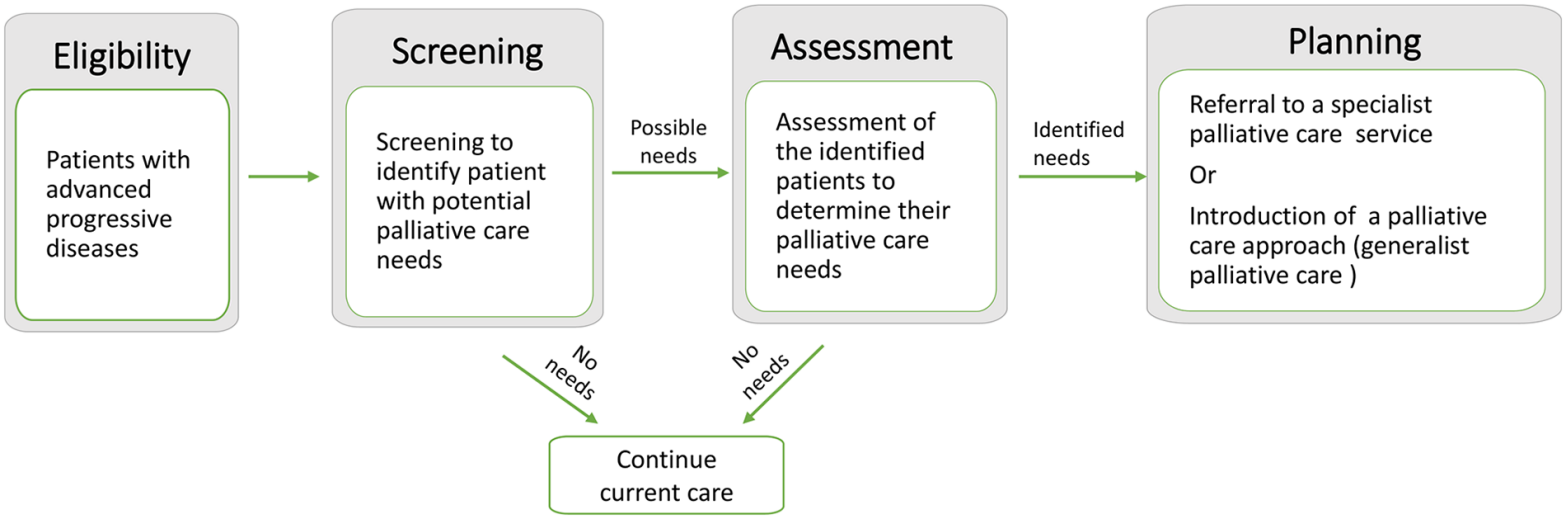
The process of patient identification and assessment of palliative care needs.

Although some of the identified tools recommended some actions to be taken after the screening process, there is no clear or appropriate care pathway for people with advanced progressive diseases who have been identified as having potential palliative care needs. Based on the findings from this review, we created a conceptual graph to describe the process of patient identification and assessment of palliative care needs (Figure 2). The first step in the process is using a screening tool to aid the identification of patients with advanced progressive diseases whose health is deteriorating and hence benefit from palliative care needs assessment. The screening tool should be based not solely on predicting mortality and deterioration but also on anticipating the needs whenever they occur and predicting the rate and course of functional decline. The identified patients who have potential palliative care needs could then be targeted for assessment to identify their unmet palliative care needs. The outcomes of the assessment can help to determine the level of care required and may prompt an introduction of a palliative care approach ‘generalist palliative care’ or referral to a specialist palliative care service.

Primary care teams play a vital role in caring for people with advanced chronic diseases.^56^ One of the main challenges for them is to identify which of their patients might have unmet palliative care needs.^57,58^ Implementing a systematic tool could help the primary care team to identify patients with advanced progressive diseases and potential palliative care needs. However, issues such as high workload and decreased resources and capacity in primary care can be barriers to implement such a screening tool.^42^ Therefore, we recommend the use of an electronic tool to systematically and automatically identify patients who might have unmet palliative care needs and trigger the use of a needs assessment tool. Although some electronic screening tools have been used such as AnticiPal and Rainoe tools, their validity is unclear as they used the risk of deteriorating and dying as a proxy for the identification of people with potential palliative care needs.^21,43,56^

The design of the future automated tools should be based on predicting functional decline and increasing needs as well as predicting mortality. Future studies of these tools should apply adequate reference standards such as palliative care interviews to examine whether the screening tools accurately identifies patients with potential palliative care needs.^50,59^ The implementation and use of these tools within current clinical practice software require minimal resources and very little training and capacity which allow them to be used in busy primary care practices.^18,60^ Implementation of validated and standardised screening tools would transform the identification process in primary care and improve timely access to palliative care for people with advanced progressive diseases and potential palliative care needs.

## Conclusion

This systematic review identified 25 studies that reported the use or development of screening tools to identify patients who are likely to have unmet palliative care needs. The evaluation of these tools was limited because of a lack of a valid comparator and so their true clinical utility is unknown. Further research is needed to identify standardised screening processes that are based not solely on predicting mortality and deterioration but also on anticipating a person’s needs whenever they occur and predicting the rate and course of functional decline in order to trigger the use of a needs assessment tool to identify and address their unmet needs at the right time.

## Supplemental Material

Supplementary_Data_FINAL – Supplemental material for Identification of patients with potential palliative care needs: A systematic review of screening tools in primary care Click here for additional data file. Supplemental material, Supplementary_Data_FINAL for Identification of patients with potential palliative care needs: A systematic review of screening tools in primary care by Yousuf ElMokhallalati, Stephen H Bradley, Emma Chapman, Lucy Ziegler, Fliss EM Murtagh, Miriam J Johnson and Michael I Bennett in Palliative Medicine
