# Supplementary material for: Identification of patients with potential palliative care needs: A systematic review of screening tools in primary care
Source: Palliat Med. 2020 Jun 7;34(8):989–1005. doi: 10.1177/0269216320929552 (PMC7388141; doi:10.1177/0269216320929552)
Supplement: Supplementary_Data_FINAL – Supplemental material for Identification of patients with potential palliative care needs: A systematic review of screening tools in primary care [file Supplementary_Data_FINAL.docx]

**Supplemental material for [Identification of patients with potential palliative care needs: a systematic review of screening tools in primary care]**

**Index**

S1: The search strategy used on MEDLINE.

S2: Bias assessment for randomized control trials (Cochrane risk of bias tool)

S3a: Bias assessment for cohort studies (Newcastle–Ottawa Scale)

S3b: Bias assessment for case control studies (Newcastle–Ottawa Scale)

S4: PRISMA checklist.

**Supplementary file 1. the search strategy used on MEDLINE.**

1. (Family adj3 Physician* or doctor* or Practi* or medicine).ti,ab.

2. (general adj3 pract*).ti,ab.

3. (GP or GPs).ab,ti.

4. (primary adj3 care).ab,ti.

5. Primary Health Care/

6. Family Practice/

7. Physicians, Family/

8. 1 or 2 or 3 or 4 or 5 or 6 or 7

9. "Referral and Consultation"/

10. Mass Screening/

11. tool*.ab,ti.

12. Risk Assessment/

13. instrument*.ab,ti.

14. diag*.ab,ti.

15. consultation.ab,ti.

16. identif*.ab,ti.

17. (case adj3 finding).ab,ti.

18. screen*.ab,ti.

19. assessment.ab,ti.

20. detection.ab,ti.

21. 9 or 10or 11 or 1214 or 15 or 16 or 17 or 18 or 19 or 20

22. Palliative Care/

23. PALLIATIVE MEDICINE/

24. Advance Care Planning/

25. TERMINAL CARE/

26. Terminally Ill/

27. Palliat*.ab,ti.

28. (terminal adj3 Care).ab,ti.

29. (advance adj3 care adj3 plan*).ab,ti.

30. (end adj3 life adj3 care).ab,ti.

31. (end-of-life adj3 care).ab,ti.

32. (terminal* adj3 ill* or patient* disease*).ab,ti.

33. ((near or approach* or close) adj4 (death ordying)).ab,ti.

34. 22 or 23 or 24 or 25 or 26 or 27 or 28 or 29 or 30 or 31 or 32 or 33

35. 8 and 21 and 34

| **Supplementary file 2. Bias assessment for randomized control trials (Cochrane risk of bias tool)** | | | | | | | |
| --- | --- | --- | --- | --- | --- | --- | --- |
| **Reference** | **Random sequence generation (selection bias)** | **Allocation concealment (selection bias)** | **Blinding of participants and researchers** | **Blinding of outcome assessment (detection bias)** | **Incomplete outcome data (attrition bias)** | **Selective reporting (reporting bias)** | **Other bias** |
| Mitchell, 2018 | Low | Unclear^a^ | *Low* | *Low* | *High^b^* | *Low* | Low |
| ^a^ Unclear allocation concealment.  ^b^ Differential drop-out rates between the two groups. | | | | | | | |

| **Supplementary file 3.a.Bias assessment for cohort studies (Newcastle–Ottawa Scale)** | | | | | | | | | | | | |
| --- | --- | --- | --- | --- | --- | --- | --- | --- | --- | --- | --- | --- |
| Domain | | Selection | | | | | Comparability | | Outcome | | | |
| **Reference** | | **Representativeness of cohort** | **Selection of non-exposed cohort** | | **Ascertainment of exposure** | **Outcome of interest** | **Comparability of cohorts** | **Assessment of outcome** | | **Adequate duration of follow-up** | **Adequate follow-up of cohort** | **Total score** |
| **Gómez-Batiste 2017,** | | 1 | 1 | | 0 | 1 | 2 | 1 | | 1 | 1 | 8 |
| **Rainone, 2007,** | | 1 | 1 | | 1 | 1 | 0 | 1 | | 1 | 0 | 6 |
| **Barnes, 2008,** | | 1 | 1 | | 1 | 1 | 0 | 1 | | 1 | 1 | 7 |
| **Moroni, 2014,** | | 1 | 1 | | 1 | 1 | 1 | 1 | | 1 | 1 | 9 |
| **Lakin, 2016** | | 1 | 1 | | 1 | 1 | 1 | 1 | | 1 | 1 | 7 |
| **Supplementary file 3b. Bias assessment for case control studies (Newcastle–Ottawa Scale)** | | | | | | | | | | | | |
| **Domain** | **Selection** | | | | | | **Comparability** | **Comparability** | | | | **Total score** |
| **Author, year** | **Is the case definition adequate?** | | | **Representativeness of the cases** | **Selection of Controls** | **Definition of Controls** | **Comparability of cohorts** | **Ascertainment of exposure** | | **Same method of ascertainment for cases and controls** | **Non-Response rate** |  |
| **Stow, 2018_b_** | 1 | | | 1 | 1 | 1 | 1 | 1 | | 1 | 1 | **8** |
| **Stow, 2018_q_** | 1 | | | 1 | 1 | 1 | 1 | 1 | | 1 | 1 | **8** |
